# Use of Notification and Communication Technology (Call Light Systems) in Nursing Homes: Observational Study

**DOI:** 10.2196/16252

**Published:** 2020-03-27

**Authors:** Haneen Ali, Huiyang Li

**Affiliations:** 1 Health Services Administration Program & Department of Industrial and Systems Engineering Auburn University Montgomery, AL United States; 2 Binghamton University Binghamton, NY United States

**Keywords:** communication systems, nursing home, response time, safety, quality of health care, observational study

## Abstract

**Background:**

The call light system is one of the major communication technologies that link nursing home staff to the needs of residents. By providing residents the ability to request assistance, the system becomes an indispensable resource for patient-focused health care. However, little is known about how call light systems are being used in nursing homes and how the system contributes to safety and quality of care for seniors.

**Objective:**

This study aimed to understand the experiences of nursing home staff who use call light systems and to uncover usability issues and challenges associated with the implemented systems.

**Methods:**

A mix of 150 hours of hypothetico-deductive (unstructured) task analysis and 90 hours of standard procedure (structured) task analysis was conducted in 4 different nursing homes. The data collected included insights into the nursing home’s work system and the process of locating and responding to call lights.

**Results:**

The data showed that the highest alarm rate is before and after mealtimes. The staff exceeded the administration’s expectations of time to respond 50% of the time. In addition, the staff canceled 10.0% (20/201) of call lights and did not immediately assist residents because of high workload. Furthermore, the staff forgot to come back to assist residents over 3% of the time. Usability issues such as broken parts, lack of feedback, lack of prioritization, and low or no discriminability also contributed to the long response time. More than 8% of the time, residents notified the staff about call lights after they waited for a long time, and eventually, these residents were left unattended.

**Conclusions:**

Nursing homes that are still using old call light systems risk the continuation of usability issues that can affect the performance of the staff and contribute to declining staff and resident outcomes. By incorporating feedback from nurses, nursing home management will better understand the influence that the perceptions and usability of technology have on the quality of health care for their residents. In this study, it has been observed that the call light system is perceived to be an important factor affecting the outcomes of the care process and satisfaction of both residents and staff as well as the staff’s performance. It is important to recognize that communication and notification technology contributes to the challenges the staff faced during their work, making their working conditions more difficult and challenging.

## Introduction

### Background

Long-term care in nursing homes provides a wide range of services for older people, especially for those who have either cognitive or physical problems, or both. The number of residents at such facilities is expected to increase from 15 million in 2000 to 27 million in 2050 in the United States [1]. As a result, nursing homes will serve an even more essential function in providing long-term geriatric services [2]. However, a recent study [3] suggests a growing concern regarding the overall safety and quality of care in nursing homes [4]. Nursing home residents usually have cognitive deficits and complex health conditions, and they often take more than one medication, which can increase the risk of medication errors [5]. According to the Department of Health and Human Services, 1 in 3 residents are harmed due to a medication error, an infection, or miscellaneous circumstances related to their treatment. These issues are widespread in nursing homes because of the lack of appropriate technologies [3,6]. Furthermore, most of the attempts to improve health care in nursing homes are solely focused on aspects such as improving medical, functional, physical, cognitive, and physiological care, in addition to providing more training for the staff [7], but not on the technological aspects.

Among the technologies used in nursing homes is the call light system, which plays a key role in communication. The call light system is critical for interactions between the nursing home staff and their residents. Research conducted in other health care settings has reported that nurse call systems significantly influence overall resident satisfaction in the delivery of their health care by creating a communication link between residents and nursing home staff [8,9]. Nurse call light systems also help to ensure the safety of patients [10]. Meade et al [10] also described it as a *lifeline* for patients because it is linked with patients’ needs and alerts the staff to the situations in which patients may ask for help. Nurse call light systems in nursing homes are associated with many ergonomic concerns and usability issues [3,6,11]. Malfunctions of the nurse call light system can cause negative medical outcomes, as the literature identifies other health care settings whereby a relationship exists between the time it takes to respond to a call light system and adverse events such as falls [12,13]. Certified nurse assistants (CNAs) contribute to more than 80% of direct care to residents. However, they must also respond to different types of alarms such as bed exit, chair exit, and clip alarms [14], as they are ultimately responsible in cases of adverse events [6]. However, their perceptions are rarely considered by the administration in the development and selection of new technology [15,16].

This study not only acknowledges the role of communication technologies as a potential solution for the challenges in nursing homes but also recognizes the importance of considering its influence on nursing practices [17,18]. This paper, therefore, provides a methodological approach to study usability challenges in the call light technologies in nursing homes. The study focuses on usability issues and challenges the staff members face while interacting and using the system to understand the barriers to productive, efficient, and safe use of these systems. The insights of the study sought to inform nursing home management on ways to improve the safety and quality of care for nursing home residents. The theoretical and methodological framework for usability evaluation is detailed in the Methods section.

### Nursing Home Challenges

The literature attributes the poor quality of care and patient safety in nursing homes to many reasons and challenges, such as lack of staff knowledge, high turnover rates, understaffing, poor management, and lack of attention to technology [19-21]. Many approaches were mentioned in the literature to overcome these problems, such as improving the quality and safety of care of nursing home residents by increasing the staffing ratio [14,20,22-24], staff training [25,26], and using technological tools such as electronic health record (EHR) and electronic medication administration [27-31]. However, no study to date has focused on the overall impact of technology on nursing homes’ work systems, and no studies have analyzed the nursing homes’ work system and how the work system elements interact with each other. All the challenges mentioned above, in addition to the lack of technology, were identified as barriers to improvement in nursing home practice [19-21]. Nursing homes need to translate the research on best practices into day-to-day practice to improve the quality of care and patient safety [19]. This can be done by analyzing the process of care and understanding the barriers to changes and how these changes will affect the outcomes of nursing homes [19].

Human factors design is increasingly recognized as an important discipline in improving health care quality and patient safety [32]. More light is being shed these days on the importance of human factors in many fields of health care, such as the usability and design characteristics of information technology systems [11,33], identifying hazards in health care [34], performance process and obstacles [35,36], and system resilience [37]. Human factors engineering approaches have also been used to analyze and describe the work system in health care settings (H Ali and A Ahmed, unpublished data, 2020), [35,38]. To overcome the challenges faced by nursing homes that impede their functionality, it is important to understand the potential benefits of technology and pay attention to how the functionality of technological innovations is improving the work system. Very few studies have examined and investigated the process of care in nursing homes or proposed approaches and practices to overcome the challenges. In addition, there is a lack of empirical studies examining technologies such as call light systems in nursing homes and their interaction with other components in the system. A thoughtful, evidence-based, and large-scale improvement that involves a new intervention is what nursing homes need to improve processes and outcomes [19].

## Methods

### Research Design

The research presented in this paper employed a cognitive engineering approach to study the interaction surrounding resident-initiated call light processes. Cognitive engineering is concerned with the analysis, design, and evaluation of complex systems of people and technology [39]. The paper presented iterative observations of continuous interaction with call light technology—beginning with the staff goal (responding to call lights in <5 min), leading to an action (being notified, locating, and addressing notifications), and resulting in a change of the system (canceling the alarm after assisting the resident). The research was conducted based on a two-pronged methodological approach to the study of human-computer interaction, qualitative research (specifically ethnography), participant observation, and interaction analysis. In stage 1, a hypothetico-deductive approach was used with unstructured observations to identify and assess the process of using the call light system. These observations took place in 4 different nursing homes to identify the challenges and usability issues associated with the different call light systems in use and to understand the barriers to productive use.

In stage 2, a detailed task analysis was used to understand and learn about the process, structure, flow, and attributes of tasks and was conducted in one of the nursing homes. The goals of this second stage were to understand the frequency, sequence, and complexity of call lights and to generate detailed and precise information on the performance of the staff and the process of being notified and responding to call lights. This stage was conducted by defining specific tasks, subtasks, and actions to be taken and by using the structured, systematic observation approach to collect the sample data.

### Procedure

Approximately 25 hours of semistructured interviews and more than 150 hours of unstructured observations were conducted by both researchers in 4 different nursing homes located in upstate New York. The goal of this stage was to understand and evaluate the different call light systems being used in nursing homes. Furthermore, the research design seemed to determine how these systems affect other elements in the nursing homework systems (person, tasks, tools and technology, organization, and physical environment), processes, and outcomes [40]. The semistructured interviews were conducted with 3 nursing home administrators, 2 unit managers, 2 registered nurses (RNs), 6 CNAs, 4 licensed practical nurses (LPNs), a program coordinator for elder service, and 1 nursing faculty with extensive experience of working in and with nursing homes. Interview topics included the following: different roles of the staff, relationships between the staff, teamwork, shifts, the technology used in nursing homes and their effectiveness, limitations, and challenges the staff experienced when using them. The study expects that how the staff is notified about call lights will affect not only their overall response time but also resident satisfaction. Furthermore, the study will identify the average time taken to respond to a call light, the effect of other alarms in the facility, and how the staff interacts and responds to the alarm. As nursing staff often considers call light notifications as interruptions, adverse events in the facility, how the staff is being notified about adverse events, delays in responding, the reasons for the delay, rounds, and how often the staff does rounds were observed to analyze how nursing home staff interacts with the call light technology (H Ali, A Ahmed, unpublished data, 2020), [9,41].

A hypothetico-deductive approach was used with the unstructured observation stage. A nonparticipant technique was employed where the observer watched from a distance for this stage of the study [42,43]. Observations were conducted in all units of the 4 nursing homes. The observation aimed to collect contextual information about the daily routines of nursing staff, the overall nursing home environment, and the tasks the staff has to do. Furthermore, the study seeks to gather information about the call light technology and other technologies that are implemented in nursing home settings and to conduct an analysis of ergonomic challenges these implementations faced (see Table 1).

**Table 1:** Methods.

Method	Aims	Setting/participants	Procedure
Interviews	To understand the routine tasks, workflow, units, incidents (adverse events), teamwork and communication strategies, technologies, strategies implemented to providing care, limitations, and challenges	Professionals from 4 different nursing homes: administrators, unit managers, program coordinators, RNs^a^, CNAs^b^, LPNs^c^, and nursing faculty	Researchers met with nursing home professionals and took notes
Stage 1 observation	To collect information about the nursing homes’ *work system* process and outcomes	Four nursing homes’ staff (RNs, CNAs, and LPNs)	The hypothetico-deductive approach was used with a nonparticipant technique. Systems Engineering Initiative for Patient Safety was used to organize the observations
Stage 2 observation (task analysis)	To observe the process of locating and responding to call lights (interaction with the technology)	One nursing homes’ staff (CNAs and LPNs)	A systematic approach observation was used, and a standard procedure of 4 steps was developed (see the Methods section)

^a^RN: registered nurse.

^b^CNA: certified nurse assistant.

^c^LPN: licensed practical nurse.

The second stage was conducted in one of the nursing homes for the task analysis. Approximately 90 hours of a structured, systematic observation approach was employed to collect data related to the process of using the call light system and to record and observe the process of locating and responding to call lights [42,43].

A standard procedure was used to observe how staff responded to call lights where the observers would stand near the nurses’ station from where they could see the system displays. The observers would take notes and use a stopwatch to record the time for each event and observe any action the staff took after a call light alarm was triggered. The study observed (1) the actual nurse response time for each call bell (the time from the initiation of the call to the time of entering the room); (2) how the nurse located the resident—using the nurses’ station display, using the light above the resident’s door, or by the resident’s verbalizations; (3) whether the CNA assisted the resident within the allotted response time (how long the resident had to wait); and (4) whether the CNA canceled the nurse call light at the time of response or forgot to cancel it— causing another CNA (or the same CNA) to respond to the same alarm. A total of 201 call light interactions were observed in approximately 3 months. The data were collected in both units: short-term care unit (south unit) and long-term care unit (north unit). The data were also collected during different time intervals, including before and after breakfast, before and after lunch, and before dinner. Of the 201 call light interactions, 97 were collected in the south unit, and 104 were collected in the north unit. Next, analysis of variance (ANOVA) was conducted to test whether the time of collecting the data (eg, before/after breakfast and before/after lunch) and unit type have a significant effect on the call light response time. Both stages were conducted after receiving the oral consent of the staff working at the time of data collection. The study was approved by the nursing homes and the university institutional review boards.

### Settings and Participants

The observations took place in 4 skilled nursing homes located in the southern tier of upstate New York. All the nursing homes included regular long-term care units and memory units (serving residents with Alzheimer’s disease and dementia or other patients deemed in need of the special care provided in such units). The number of beds in the facilities ranged from 150 to 381. Moreover, 2 of the facilities were private care units, 1 was public (by county), and a nonprofit organization ran the fourth facility. In total, 150 hours of observations were conducted during the 3 different shifts (morning: typically, 7 AM-3 PM; afternoon: 3 PM-11 PM; and night: 11 PM-7 AM). The participants in this study were nursing home staff and residents. CNAs and LPNs working in the different units of the nursing homes were all participants. Residents of nursing homes were also observed to study how they influence interactions with different technologies. The task analysis stage was conducted in 1 public 300-bed nursing home. This nursing home was chosen for this stage because there were many complaints from residents and residents’ families about long response times and because of accessibility to the research team.

**Figure 1:**
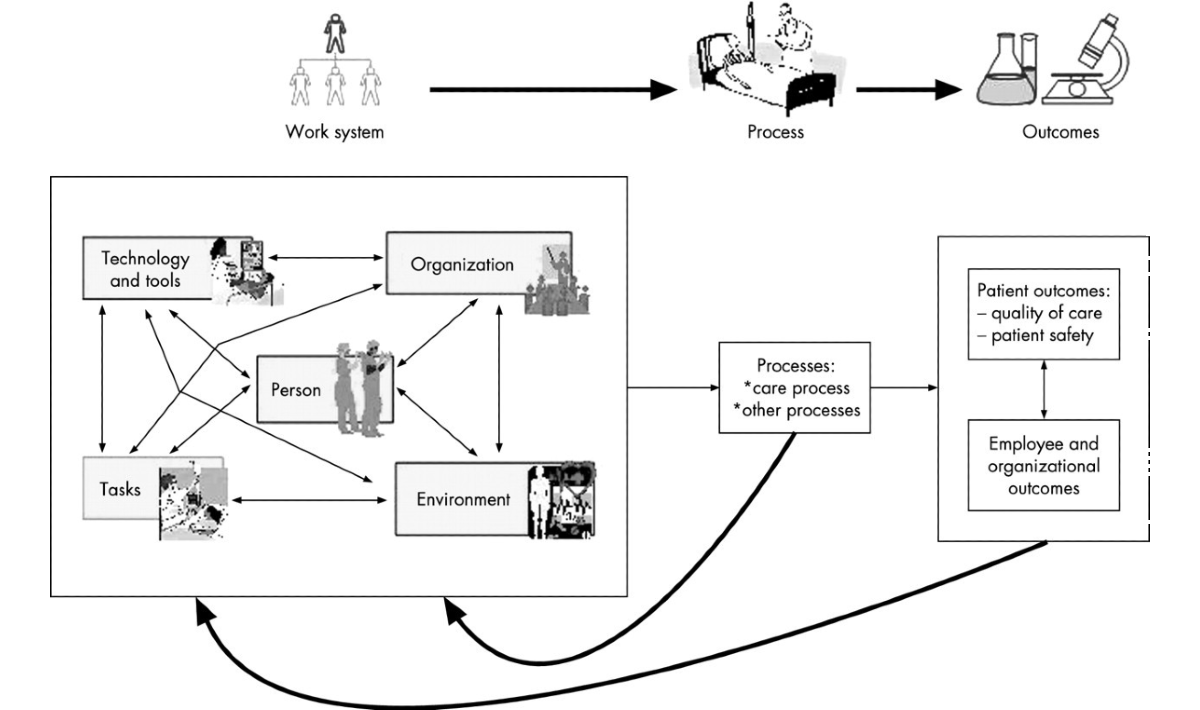
Systems Engineering Initiative for Patient Safety model of work system and patient safety.

All relevant aspects of observations were categorized into groups and subgroups using the Systems Engineering Initiative for Patient Safety (SEIPS) model of work system and patient safety as a guide [40] (Figure 1). Observations about behavior, linguistic aspects (eg, speech), extralinguistic aspects (eg, sounds/loudness), and staff’s and residents’ comments were all coded and categorized using the SEIPS model. Moreover, 2 of the researchers categorized the interviews and recorded observations individually. Finally, multiple focus sessions were conducted to discuss any disagreements on the placement of notes into the main categories.

## Results

### Work System

#### Staff

Nursing home staff, the primary drivers of care and daily treatments, are essential people at a nursing care facility. RNs are usually the unit managers or supervisors. They graduate from a state-accredited nursing program and have been licensed by the State Board of Nursing. They are the ones responsible for residents’ care plans and the ones who assess residents in case of a fall or any other type of injury. They are also the ones to start and manage intravenously and administer any medication by vein. LPNs were originally educated and trained to work as bedside nurses in hospitals and are now working in nursing homes, rehabilitation centers, physicians’ offices, schools, and clinics. Becoming a licensed practical or vocational nurse requires the completion of a formal training program plus supervised clinical instruction. They all have high school diplomas or their equivalent and have graduated from a program with a license granted by the State Board of Nursing. Their main task is to administer medication and take vital signs, but they are also required to respond to the various alarms in the unit. LPNs work under the close supervision of RNs. CNAs completed a training program at a state-approved facility. They provide most of the direct care and assist residents with their daily activities such as toileting, shower, changing clothes, transportation, and mealtime.

#### Organizational Characteristics

Organizational policies and standards are also important to keep residents safe. According to nursing home administrators, all staff working in the unit are required to respond to all bed and chair exit alarms and must respond to the call lights within 5 min. It was observed that LPNs often ignore call light notifications, in many cases, walking past residents in need. It was also observed that it takes more than 5 min to respond to a call light; in some cases, these responses can take up to 20 min.

In many cases, the staff enters the resident’s room to ask about their needs and asks them to wait and forgets to come back, in which case, the resident had to use the call light again (see the Processes section). Many complaints were observed in one of the nursing homes regarding the long response time. Nursing home administration was considering the option of upgrading their current call light system to a system capable of monitoring response times of the staff. This information would be used to reduce the number of complaints received by reporting response times to the residents’ families to assure families that the response time is within an acceptable range.

Communication across the staff working in the same unit is important for the safety of residents. However, there is no system for the staff to communicate in the same unit or between the staff in different units. If 1 CNA was busy with a resident and another resident needed help, the CNA could not leave the resident to assist another resident. In addition, CNAs, in some cases, cannot provide care to residents without having 2 people involved (eg, using a lifting machine). With the lack of communication systems, it is often difficult for the CNAs to find help; as a result, many CNAs resort to running across the unit to find an available CNA or LPN. This can cause a delay in assisting residents and might lead to an adverse event. Vertical communication within a working system is also important. CNAs have reported limited communication with the RNs on the unit because of their high workload.

Between 2 shifts, there is a 30-min overlap. The second shift, for example, starts at 2:45 PM, whereas the first shift ends at 3:15 PM. During this overlap, staff members are supposed to hand off reports (CNAs to CNAs and LPNs to LPNs) and to make their rounds. Rounding is a means of transferring information to the staff coming on shift and giving them updates regarding the residents’ current health care plan. Rounding usually takes place during the overlap between shifts. Staff members are supposed to check safety mechanisms during rounding and report any changes in a resident’s condition, such as changes that might require immediate attention and responses to call lights from these residents. However, the checklist is not always followed. Normally, only 2 to 3 people perform their rounds because the others are busy with tasks such as charting. CNAs, who are typically the busiest, tend to underestimate the importance of rounding and do not do it. A lack of direct communication between the 2 shifts was observed, which might result in the lack of feedback regarding the residents’ conditions.

#### Tasks

Residents’ rooms are divided into sections, and certain rooms are assigned to teams. This assignment remains unchanged for the whole month to ensure the consistency of service provided to residents. Each CNA oversees 8 to 13 residents, and each LPN oversees 9 to 14 residents. There are 3 shifts per day with 8 hours each. Day shifts have a higher level of staffing than evening and night shifts.

As a policy, when an alarm goes off, whether it is a call light, chair exit alarm, bed exit alarm, or a clip alarm, everyone is expected to respond, including housekeepers. There might be multiple staff members responding to an alarm until the assigned team is assisting the resident. Staff members communicate primarily in person. For example, 2 staff members may go to the same room to address an alarm where they may also discuss another ongoing alarm and notify each other about their plans to deal with it.

Even if the staff is notified on time, they may not be able to address the resident’s needs on time. For example, if they were occupied with someone else, they cannot leave that resident until all scheduled tasks related to the resident’s treatment and safety have been completed. By the time a staff member reacts to the alarm, the patient may have already fallen [5,17]. This issue is more significant in the morning. Most residents wake up between 6:45 AM and 7:45 AM. Numerous alarms can go off simultaneously when they all try to get out of bed or begin moving at the same time. The staff members must race from one room to another; they rely on their experience and try to start with residents who cannot walk or stand by themselves. However, there are typically more residents needing assistance than available CNAs during these times. At one instance, CNAs asked the observers to watch residents several times that day because of their need to stay with their current resident and the lack of available staff members to help.

The noise from the many alarms can also confuse the staff, as the noise produced from the auditory systems is almost constant. This causes discomfort for both residents and staff members. Alarm fatigue can also develop over time because of the nursing staff receiving too many nonurgent call lights [42]. Throughout their careers, nurses learn how to prioritize tasks because of the need and the time allotted in their shift. As nurses are unable to distinguish urgent alarms from the call light from the nonurgent, calls associated with the call light tend to come from nonurgent needs. This leads the nursing staff to assume that residents do not need urgent help. As nurses begin to feel more confident in how they assess patients and understand their acuity and treatment, they also learn how to work without relying on the call light. This is the beginning of what develops into alarm fatigue, as learning how to work without relying on the system can cause nurses to ignore the alarms. Although using a pager can reduce the noise and display the room number, because of the fast-pace routine performed by nursing home staff members, most are too busy providing round the clock service for residents to grab the pager and read the room number.

Nursing homes are often understaffed, and nurses’ stations may not always be occupied. As nursing staff is always on the move to care for residents, this makes using a centralized monitoring system difficult. Owing to the tight schedule and understaffing, although CNAs are working with residents, they may not be able to provide time for rounding as planned. As a result, residents with risks may not be checked as often as prescribed. CNAs must either stay late after their shift is over to enter updates to the EHR system or use their breaks and become exhausted because of working up to 12 hours without a pause.

In addition, a single staff member may be assigned to multiple residents at the same time, and this can be particularly stressful when more than one resident needs attention, such as instances whereby more than one resident must be transported. Although working on their own, these logistical hurdles can create chaotic moments for both the nursing home staff and their residents. Further details are included in the summary of observations in Multimedia Appendix 1.

#### Tools and Technologies

A total of 3 different call light systems were observed. Included in each system were nurse station consoles, which triggered an alarm to indicate a call light. All systems have lights placed above the residents’ doors, and the lights come in 2 colors (white and either red or orange). These lights help in locating the senior using the call light. The white light indicates that the resident is asking for assistance from the bedroom, whereas the red or orange light indicates that the resident is asking for assistance from the bathroom.

The first call light system did not display the room number; instead, it only triggered an auditory alarm that is also broadcasted over speakers throughout the units. The auditory alarm uses a series of *beeping* at 2 levels of speed: slow beeping indicates that the call is coming from the bedroom, and fast beeping indicates that the call is coming from the bathroom. At the ceiling above the nurse station, there is a group of 4 lights, which indicates 4 different areas in the unit. These lights turn on in response to a call light. The staff must locate the area first and then locate the resident by identifying the lights above the doors. If the lights for multiple areas are on, then the staff must check the lights above the doors in those areas.

The second call light system observed was also used at the nurses’ station console. The console could display the room number to help the CNA locate the room easily; however, it could only display 1 room number at a time. If a new alarm were triggered, it would override the previous room number. In this system, the only auditory warning triggered is at the nurses’ stations. If the staff was working down the hallway or inside the residents’ rooms, they could not hear the alarm. Furthermore, there is no auditory distinction between a call bell in the bedroom or the bathroom, with the latter often associated with more urgent needs. It was observed that the console was muted in 1 unit and was covered with a file in another unit in the same nursing home.

The third call light observed was a pager-based system that displays the room numbers and emits an auditory *beeping* sound. In this system, the nurse station console can also display the room number and emit an auditory alarm. The system, however, can only display 1 room number at a time. In cases of more than 1 alarm, the console switches between the room numbers, displaying each room number for approximately 5 seconds before switching to display the next room number. To clear the override, the staff must cancel the alarm from inside the resident’s room and from the pager (Multimedia Appendix 2).

Call light systems include visual and auditory alarms. The visual alarms may indicate a location, whereas the auditory alarms are not directional, as the sounds are emitted through loudspeakers located along the hallway walls or broadcasted directly from the nurse station.

#### Usability Issues of Call Light Systems

There are many usability issues in the current call light system. First, the staff is often unable to find a break in their tasks to contribute to monitoring the central display of the call light. Some systems could only handle 1 alarm at a time. If 2 or more residents triggered an alarm, room numbers would not display until the first one was resolved or the system would display the newest alarm and cover the previous one. In both cases, the nursing home staff have no access to feedback information about the number of alarms in the unit, if the alarms were resolved, and whether the residents were properly assisted. Not distinguishing between the alarms from bedrooms and the alarms from bathrooms (which often reflects more urgent needs) is another usability issue. In addition to reporting to the nurse station to receive the notification, the staff must also look down the hall to distinguish the alarms by using the lights above the residents’ room doors.

Furthermore, the nursing home staff are working inside the resident’s room most of the time. As a result, they lack access to hear the alarm when the system alerts them from the nurse station. In addition, systems that broadcast alarms using loudspeakers and other alarms in the unit constantly adjust the noise level in the nursing home to a high pitch. Although this is not desirable for the nursing home staff, it is even worse for the residents; the response time to call lights is long because the nurse station is not staffed most of the time. Using a pager can reduce the noise and display the room number, but the nursing home staff is always busy providing service for residents, and they cannot grab the pager to see the room number. Furthermore, the auditory sound is not directional and, thus, is unhelpful for locating the resident’s alarm. As a result, many staff members mute their pagers or leave them at the nurse station. CNAs tend to locate the room by looking at lights on the top of the doors, which can also be challenging at times because of the layout. The timing of the alarms also causes problems. Usually, when the alarm goes off, the patient may have already fallen. For example, a resident who tries to stand up but is unable to support himself or herself may lean forward and fall.

Broken parts were also the main usability issue impeding the systems. In some units, there were many broken and nonworking lights. When a resident pushed the call light button, the auditory sound would be triggered, but the light might not have worked. In some cases, because the systems were very old, the lights would turn on, but no auditory alarm would be heard (see the summary of usability issues in Multimedia Appendix 2).

#### Physical Environment

The unit layouts for the facilities observed in this study were L- or T-shaped with 1 nurse station. Residents who have high risks or more critical conditions stay in the rooms closest to the nurse station.

The auditory alarms are active for most of the day. This can be obnoxious to live-in residents, as it often disrupts their rest and distracts them from their activities. The study finds that this ultimately works against the nursing home’s goal of creating a comfortable environment.

The floor layout and other aspects of the physical environment are poorly designed, making it difficult for the staff to know where an alarm (eg, a call bell) is signaling danger. The auditory alarms were broadcasted without any directional information. Beams and doorframes blocked certain alarms from being seen. Furthermore, door lights did not always work, which required staff to walk to the middle of the hallway. More than 90% of times, CNAs tended to locate the room by looking at lights on the top of the door; they have to walk to the middle of the hallway to see the light, which contributes to additional workload caused by usability issues.

Having many auditory notifications in nursing homes, such as bed mats, chair mats, call light systems, and the Wander Gard system, makes nursing home environments noisy and uncomfortable for many residents to live in. During our observations, many residents complained about constantly hearing the notifications and alarms throughout the day. Consequently, the staff members sometimes had to mute the call light system in some units, which posed challenges for notifying and responding to a call light. The impact of this noisy environment is not confined to residents but also reaches and affects the staff’s performance (see the summary of the observation in Multimedia Appendix 1).

### Processes

The task analysis methodology was used to analyze the task of being notified and responding to the call light system and the actions the staff took to finish the task.

The results showed that the highest response time was before dinner, and this was true in both units. ANOVA results showed that the unit type had no significant effect on overall response time (*P*=.85). However, timing has a significant effect on the overall response time (*P*=.01), whereas the interaction between the unit type and the time interval had no significant effect on the response time (*P*=.90; ANOVA table provided in Multimedia Appendix 3). In the later analysis, the response time data in both units were combined because of there being no significant difference between them.

According to the collected data, the average response time to a call light in both units was 9 min, and these responses were the longest before dinner time. Responses to call lights in the morning were also long; around 50% of the time, CNAs responded after 5 min, which exceeded the administration’s expectations or standards.

The staff did not use all the features in the system because of usability issues, as the console was muted in the south unit. CNAs had to track the lights above the rooms’ doors all the time. More than 16% of the time, CNAs forgot to cancel the alarm after they responded in this unit because there was no auditory alarm. This could cause redundancy of work if another nurse noticed the light and responded to the same resident. More than 80% of the time, CNAs were notified by the light. All the cases when CNAs were notified by the display or console took place when they were near the nurse station where they could hear and see the display. In more than 7.5% (15/201) of the cases, because of broken parts in the system, CNAs were notified by the residents. After pushing the button for assistance, if no one responded to them, residents tended to stand and attempt to help themselves or to go the room door to look out and shout angrily for a nurse’s attention. In more than 10.0% (20/201) of call lights, CNAs responded and asked the resident to wait until they were done with someone else. Around 3% of the time, they forgot to come back to assist the first resident.

## Discussion

### Communication in Nursing Homes

Approximately 34,000 fatal, life-threatening, or serious adverse events per year occur in nursing home settings, and most of these events are considered preventable [44,45]. A lack of effective communication policies at all levels of nursing homes was found to have contributed to these events [44,46,47]. Research in other health care settings suggests that the call light system is the link between nurses and patients during hospital stays [13]. It is also recognized as a crucial piece of technology for patient-centered care models [48]. It was also found to highly influence the satisfaction levels of nursing home staff and patients [8,9] and highly affect the safety of patients [10,49].

In nursing homes, the call light system is the means of the initial communication between staff and residents. We endeavored to understand how nursing home staff use the system by observing their interactions and identifying the usability issues associated with the call light systems that prevent the staff from providing quality health care.

### Principal Findings

In this study, it was observed that nursing home staff that interact with the call light systems are often CNAs. However, according to nursing home administrations, all staff are responsible and supposed to respond to all types of alarms in the unit, such as bed or chair exit, clip alarm, weight sensors, and call light. Device alarms are intended to alert and inform nursing home staff of any changes in the residents’ conditions. However, the staff is subjected to too many alarms that disturb their workflow and might lead to many errors, which can contribute to mistrust and long response times [42,50]. CNAs provide most of the direct care to residents and respond to most of the alarms in the unit. However, around 50% of the time, they responded after 5 min, which exceeded the administration’s expectations and standards. A high alarm rate limits CNAs’ ability to manage the call system properly, resulting in negative perceptions and alarm fatigue by nursing home staff. Having a high number of alarms can cause the staff to ignore the call bell occasionally. The staff was notified about call lights by the residents themselves. Around 7.5% (15/201) of these instances involved the residents getting out of their bed or chair and going to the room door to ask for help. The study finds that this is because the CNAs hold negative perceptions of residents’ use of the call light because of frequent, nonurgent use. This ultimately causes misconceptions about the purpose of call lights because of their frequent use [51]. It was also observed that staff members often muted the call light system in some units, which posed a challenge for proper notification and increased the response time to a call light. Many broken parts were observed in 3 facilities, and this may have caused malfunctions in the call light system, resulting in adverse events and contributing to longer overall response time. These delays in responding ultimately increased the risk of residents harming themselves because of an unanswered alarm.

The physical environment of nursing homes was also found to be an issue. Nursing homes attempt to create a home-like environment; however, they are designed in hospital layouts, with L- or U-shaped units and with multiple bedrooms located on long double-loaded hallways. The call light system has one console placed at the nurse station in the units that are unattended most of the time and lacks any directional information. In more than 70% (86/201) of the observed interactions, the staff located the alarms using the lights above the doors because the consoles were not visible or accessible to them. This required that they had walked out of a resident’s room or away from the nurse station. In addition, CNAs are spending most of their time in residents’ rooms providing care, and as a result, they are oblivious to the alerts of the call light, console, and auditory alarm.

Understaffing is one of the main problems in nursing homes. Thus, the design of technology should try to overcome this problem by providing solutions that are less staff dependent or require less effort from the staff. Due to understaffing, protocols such as rounding may not be followed strictly. Thus, the system should provide important information redundantly to ensure it is received by staff.

The staff did not use all features of the call light systems because of usability issues. CNAs had to look and keep track of the lights all the time. More than 16% of the time, CNAs forgot to cancel the alarm after they responded because there was no auditory alarm because of broken parts or because of continued auditory alarms in the unit. This could cause redundancy of work if another nurse noticed the light and responded to the same resident. More than 80% of the time, CNAs were notified by the light. All the cases when CNAs were notified by the display or console took place when they were near the nurse station area where they could hear and see the display. In many cases, because of broken parts in the system, CNAs were notified by the residents. After pushing the button for assistance, if no one responds to them, they tend to stand to help themselves or go the room door to look and shout angrily asking for help.

In many cases, CNAs had to prioritize when more than one call light was triggered simultaneously or when the interval between call lights was only slightly different. Sometimes, they responded by asking the residents to wait until they were done with someone else. Around 3% of the time, they forgot to come back to assist the first resident. This is because of the staff’s high workload and busy schedule [14,52], which might also contribute to the long response time and not following protocols such as rounding and muting the system.

Our analysis of the call lights in nursing homes showed that most call lights were used before and after mealtimes. The average response time to a call light was 9 min, and the wait was the longest before dinnertime. This is because residents were tired at the end of the day and were ready to sleep. Response time was also long in the morning because residents tend to walk up at the same time, and everyone needs to get to the bathroom, get dressed, and have breakfast.

### Conclusions

In conclusion, the call light system is critical for interactions between the nursing home staff and residents. Research conducted in other health care settings has demonstrated that the call light system not only significantly improves the communication between staff and patients together but also helps ensure the safety of patients. Nursing homes still using old call light systems risk the continuation of usability issues that can affect the performance of the staff and contribute to a decline in staff and resident outcomes. Although the health care industry has been at the forefront of technological advancements and implementation, it is important to recognize the importance of considering the perceptions of end users in the development of new medical technology and the overall quality of health care service delivery. By incorporating feedback from nurses, nursing home management will better understand the influence that perceptions and usability of technology have on the quality of health care for their residents. In this study, it has been observed that the call light system is perceived to be an important factor affecting the outcomes of the care process and the satisfaction of both residents and staff as well in addition to the staff’s performance. Staff shortage, mental and memory conditions of residents, usability issues associated with the call light system, and nursing home layout can also contribute to long response time, which might affect the safety of the residents of nursing homes.

The insights from this work were used to design a smart notification and communication system for nursing homes [3]. The smart system was designed to overcome the challenges the staff members face and aimed to improve their performance. In addition, the effects of a call light system on nursing homework system, the staff’s perception about the call light system, and the process of responding to a call light will be thoroughly investigated in a future publication for more understanding and evaluation of the contribution of a call light system to safety and quality of care in nursing homes.

## Appendix

Multimedia Appendix 1 Summary of observations.

Multimedia Appendix 2 Description of the call light systems observed and the usability challenges associated with each system.

Multimedia Appendix 3 Analysis of variance.

## Abbreviations

ANOVA: analysis of variance
CNA: certified nurse assistant
EHR: electronic health record
LPN: licensed practical nurse
RN: registered nurse
SEIPS: Systems Engineering Initiative for Patient Safety
