# Supplementary material for: Use of Notification and Communication Technology (Call Light Systems) in Nursing Homes: Observational Study
Source: J Med Internet Res. 2020 Mar 27;22(3):e16252. doi: 10.2196/16252 (PMC7148550; doi:10.2196/16252)
Supplement: Multimedia Appendix 1 [file jmir_v22i3e16252_app1.pdf]

Summary of observations

Components of the Systems Engineering Initiative for Patient Safety model	Summary of observations
Person	• Most of the staff are female<br>• Staff<br>○ *RNs*[a]: roles include creating nursing assignments, making up the working schedules, developing resident's treatment plans, administering medicine (Intravenous (IV) and blood) in addition to their supervisory role (primary care managers)<br>○ *LPNs*[b]: roles include directing bedside care, checking vital signs, administering medication, collecting samples for testing, feeding patients who use feeding tubes, and performing other procedures<br>○ *CNAs*[c]: roles include routine tasks in the provision of personal care to residents, such as eating, dressing, bathing, and getting in and out of bed<br>• Unit: long-term care and short-term care unit.<br>○ Three shifts<br>• Other: demonstrates a lack of teamwork and poor working conditions
Organization	• Incompliance with protocols: overruling rounds and muting the system<br>• LPNs tend not to respond (all staff must respond to alarms), the long response time (more than 5 min to respond to a call light), lack of collaboration (teamwork and difficulties in

	finding help) and communication, and the nurse station are understaffed<br>• Poor maintenance of the system: many broken parts<br>• Control over the practice and supervisor support:<br>○ The schedule is flexible to some degree<br>○ There are limitations in providing input/suggestions, staff shortage, and blaming staff in case of adverse events such as falls
Task	• Responding to a call light: all staff are expected to respond within 5 min<br>• High mental and physical workload: information processing and memory (divided attention and multitasking: slower and less accurate tasks and change blindness), interruptions, diagnosticity (staff perceptions about call lights), and alarm fatigue
Environment	• Layout: not visible (display at nurse station), lights above the resident’s rooms are not visible (blocked by beams and doors), and long hallways (hard to hear)<br>• Noise: loudspeakers to broadcast alarms; the alarm is on most of the time, residents complain about the noise
Technology/tools	Usability issues<br>• The nurse station is not occupied<br>• No directional information<br>• There is a lack of prioritization and low/no discriminability; is it a bathroom or bedroom<br>• Many broken parts (can cause failure to detect changes)<br>• The location/position of the display (not accessible)

	• The staff can mute the system<br>• The system handles only 1 alarm at a time; new alarm "overwrites" previous alarm<br>• There is no feedback (process to monitor) about the previous alarm<br>• False alarms<br>• Loudspeakers (noise)
Process	Call lights (alarms) notification and location process
Outcomes	Staff overall job satisfaction, stress, and burnout

[a]RN: registered nurse.

[b]CNA: certified nurse assistant.

[c]LPN: licensed practical nurse.
