# Supplementary material for: Use of Notification and Communication Technology (Call Light Systems) in Nursing Homes: Observational Study
Source: J Med Internet Res. 2020 Mar 27;22(3):e16252. doi: 10.2196/16252 (PMC7148550; doi:10.2196/16252)
Supplement: Multimedia Appendix 2 [file jmir_v22i3e16252_app2.pdf]

Description of the call light systems observed and the usability challenges associated with each system.

Call light system type	System description	Usability issues and challenges with the system
Traditional/type 1	• Two lights above residents' room<br>• One console without a display at the nurse station<br>• Four lights at the ceiling above the nurse station area to indicate 4 different areas in the unit<br>• Auditory alarms are broadcasting at a nurse station in addition to multiple locations in the unit<br>• Two speeds of auditory alarms—slow beeping for bedrooms call and fast beeping for bathroom calls<br>• One nurse station in a U-shaped unit	• -Nurse station is not occupied; no one to monitor the console/central display<br>• Low/no discriminability<br>• Lack of prioritization<br>• If more than 1 area light is on, staff must check all these areas<br>○ Noise; load speakers<br>○ False alarms because of broken parts<br>○ Lack of feedback<br>○ Noise<br>○ Lights above the resident's rooms

	• Alarms can be canceled from residents' rooms	are not visible because of layout
Traditional/type 2	• Two lights above residents' doors<br>• Console with a display at the nurse station<br>• Auditory alarms are broadcast at the nurse station<br>• One nurse station in a T-shaped unit<br>• Alarms cannot be heard from inside rooms at the end of the hallways<br>• No sound differences between alarms from bedroom and bathroom<br>• Staff can mute the console<br>• Alarms can be canceled from residents' rooms	• The nurse station is not staffed<br>• The console can only display 1 room number<br>• Low/no discriminability; is it a bedroom or bathroom<br>• Lack of prioritization<br>• Many broken parts/false alarms and redundancy of work<br>• Console and auditory alarms are not accessible from residents' room and end of the hallways<br>• The new alarm ”overwrites” previous alarm<br>• The lights above the resident's rooms are not visible because of layout

		• The staff can mute the system
Pager-based system	• Console with a display at the nurse station<br>• The console can only display 1 room number and will keep switching between different room numbers in case of more than 1 alarm<br>• Auditory alarms are broadest on the nurse station in addition to a beeping sound from the pagers<br>• Alarms must be canceled from both the residents' rooms and the pagers	• The nurse station is not staffed<br>• There is a lack of directional information. Console switches between room numbers every 5 seconds, and staff might cancel the wrong alarm<br>• Pagers are muted most of the time<br>• The staff has a busy schedule and cannot grab the pager every time they have an alarm<br>• Certified nurse assistants must make trips to the nurse station to cancel the alarm, and alarms must be canceled from the pager and the console/center display

		• The lights above the resident's rooms are not visible because of layout<br>• Staff might take a pager that is not for them (they take the pager at the beginning of the shift)
