# Supplementary material for: Use of Notification and Communication Technology (Call Light Systems) in Nursing Homes: Observational Study
Source: J Med Internet Res. 2020 Mar 27;22(3):e16252. doi: 10.2196/16252 (PMC7148550; doi:10.2196/16252)
Supplement: Multimedia Appendix 3 [file jmir_v22i3e16252_app3.pdf]

Analysis of Variance

Source	DF	Adj SS	Adj MS	F-Value	P-Value
Unit Type	1	0.0134	0.01343	0.04	0.848
Time Interval	4	5.0157	1.25392	3.44	0.010
Unit Type*Time Interval	4	0.3907	0.09768	0.27	0.899
Error	191	69.7054	0.36495		
Total	200	75.5656			
